# Latent Cytomegalovirus Reactivation in Patients With Liver Failure: A 10-Year Retrospective Case-Control Study, 2011-2020

**DOI:** 10.3389/fcimb.2021.642500

**Published:** 2021-05-10

**Authors:** Qingluan Yang, Zhe Zhou, Xuefang Yang, Yuming Chen, Aiping Liu, Bingyan Zhang, Lingyun Shao, Jianming Zheng, Wenhong Zhang

**Affiliations:** ^1^ Department of Infectious Diseases, Huashan Hospital, Fudan University, Shanghai, China; ^2^ Department of Infectious Disease, The Second People’s Hospital of Lanzhou, Gansu, China; ^3^ Clinical Laboratory, Huashan Hospital, Fudan University, Shanghai, China; ^4^ National Clinical Research Center for Aging and Medicine, Huashan Hospital, Fudan University, Shanghai, China; ^5^ Key Laboratory of Infectious Diseases and Biosafety Emergency Response, Huashan Hospital, Fudan University, Shanghai, China; ^6^ State Key Laboratory of Genetic Engineering, School of Life Science, Fudan University, Shanghai, China; ^7^ Key Laboratory of Medical Molecular Virology (MOE/MOH) and Institutes of Biomedical Sciences, Shanghai Medical College, Fudan University, Shanghai, China

**Keywords:** cytomegalovirus, reactivation, liver failure, glucocorticoids, mortality

## Abstract

**Background:**

The aim of this study was to explore potential risk factors for cytomegalovirus (CMV) reactivation and their impact on liver failure patient outcomes.

**Methods:**

A 10-year retrospective case–control study was conducted in adult participants, who were diagnosed with liver failure and had undergone CMV DNA tests. CMV reactivation cases were matched with controls at a 2:1 ratio based on age, sex, and year of admission. Univariate and multivariate analyses were used to explore risk factors for CMV reactivation.

**Results:**

Between January 2011 and April 2020, 198 adult patients with liver failure and available CMV DNA test results were enrolled into the study. Among them, 33 patients had detectable CMV DNA in their plasma (16.7%). Clinical manifestations and liver function were comparable between the CMV reactivation and non-reactivation groups. However, CMV reactivation may triple mortality in patients with liver failure. We found that nearly 50% of patients in the CMV-positive group received glucocorticoids, compared to 13.6% in the CMV-negative group (*P*=0.000). The median total glucocorticoid dose included 836.5 mg of methylprednisolone (IQR 308.7-1259.0 mg) in the CMV-positive group, which was significantly higher than that in the CMV-negative group. A multivariate analysis revealed that glucocorticoid use significantly increased the risk of CMV reactivation (adjusted OR, 4.84; 95% CI, 1.61–14.49; *P*=0.005). Patients with CMV reactivation tended to be associated with higher white cell counts (adjusted OR, 1.21; 95% CI, 1.08–1.36; *P*=0.002).

**Conclusions:**

High intravenous glucocorticoid doses may be the most important risk factor for CMV reactivation in liver failure.

## Highlights

CMV reactivation was associated with mortality in liver failure and high glucocorticoid doses intravenously seemed to be the most important risk factor for CMV reactivation in liver failure. Thus, monitoring CMV DNA dynamically may be considered when using glucocorticoid.

## Introduction

The Cytomegalovirus (CMV) is a common β-herpesvirus that establishes lifelong latency following primary infection. After initial infection, CMV persists within the body. The CMV seroprevalence rate in healthy adults reportedly ranges from 40% to 100%, whereas the number in the Chinese population is nearly 100% (Cannon et al., 2010; Marsico and Kimberlin, 2017). Typically, immunity against the virus controls its replication, although intermittent viral shedding can continue to occur in a seropositive immunocompetent person (Gandhi and Khanna, 2004). However, in immunocompromised populations, CMV has become one of the most important opportunistic pathogens, such as in patients undergoing organ transplantation or hematopoietic stem cell transplantation (HSCT), who have the highest risk of developing CMV-associated complications (Singh et al., 2020). CMV diseases exist in a wide spectrum, ranging from asymptomatic viremia to severe systemic and gastrointestinal tract disease, which has been regarded as a major cause of morbidity and mortality in immunocompromised populations (von Muller et al., 2007; Berman and Belmont, 2017).

Systemic inflammation is a key feature of liver failure, and poor disease outcome is closely associated with exacerbated systemic inflammatory responses (Clària et al., 2016). Similar to sepsis, a cytokine storm can occur in liver failure, especially in acute-on-chronic liver failure (ACLF). Owing to associated immune disorders, coinfections with other typical and opportunistic pathogens may be common and related to liver failure prognosis. Previous studies indicated that CMV might be reactivated in patients with cirrhosis, thereby inducing fulminant hepatic failure (Arai et al., 2009; Hsu et al., 2014; Rosi et al., 2015). While numerous studies have reported CMV in patients undergoing organ transplantation or HSCT, few studies are available regarding CMV infection in patients with liver failure (Cho et al., 2019). A retrospective study conducted in China analyzed the risk factors for both CMV and Epstein-Barr virus infection and their influence on the liver function of patients with ACLF-based hepatitis B virus, indicating that 5% (5/98) of the patients presented with CMV DNA (Hu et al., 2018). However, the study was conducted on a small scale, and the influence of prognosis on CMV infection was not confirmed. Currently, the full spectrum of CMV disease, risk factors for reactivation, and long-term clinical implications are not well understood in patients with liver failure. An improved understanding of CMV reactivation in patients with liver failure is important to elucidate the role of virologic surveillance, prophylaxis, and antiviral therapy.

The aim of this study was to describe a 10-year retrospection of patients with liver failure and CMV reactivation and to explore potential risk factors for CMV reactivation and impact on outcome.

## Materials and Methods

### Study Design and Participants

**Figure 1:**
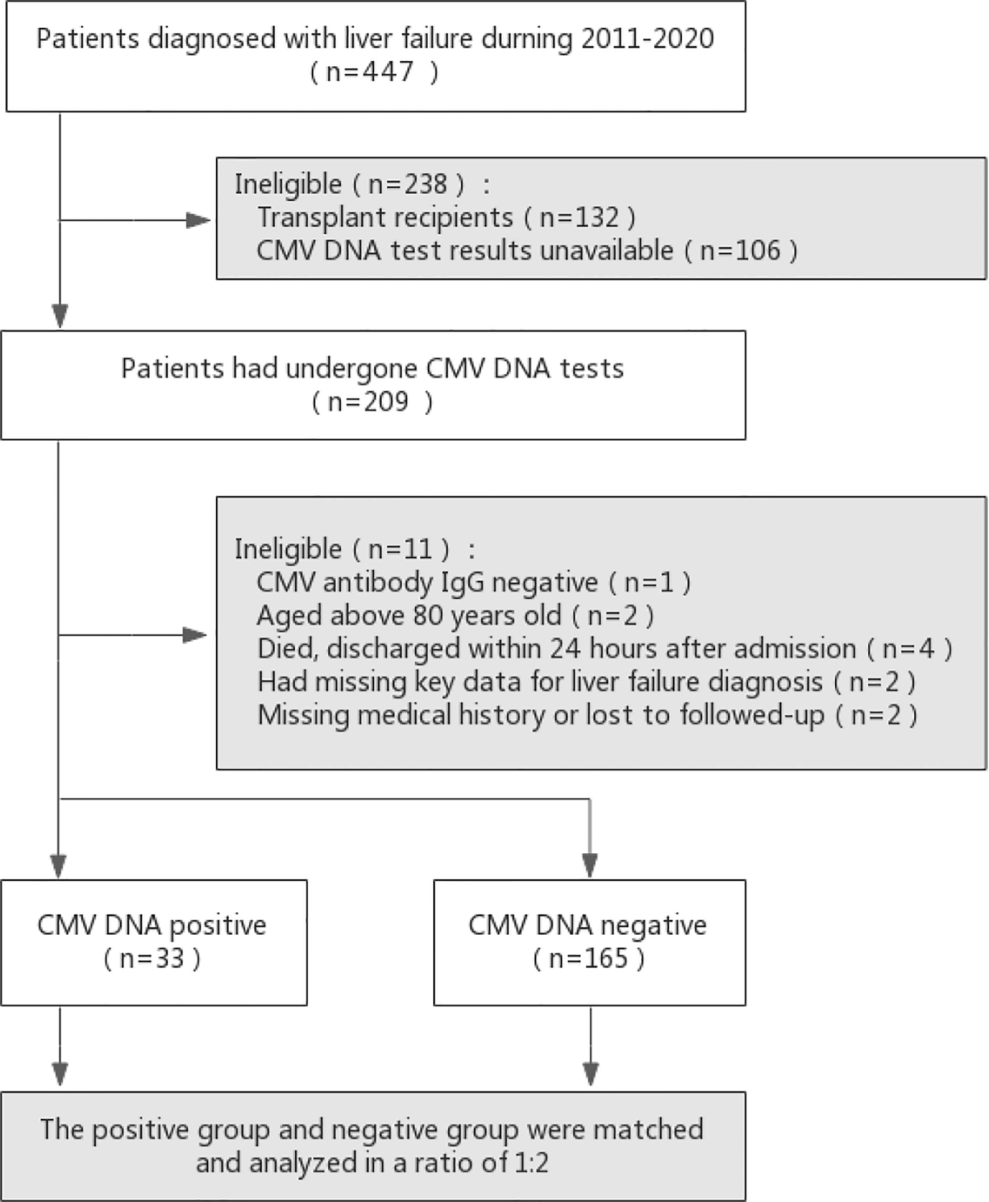
Flow diagram of patients included in the study. Potential controls were matched to cases based on age (within 8 years) and year of admission (as close as possible, but no more than 1 year before or after).

This retrospective case–control study was conducted in adult participants in the Department of Infectious Diseases at Huashan Hospital affiliated to Fudan University in China. To explore the reactivation of latent cytomegalovirus infection in patients with liver failure, we retrospectively evaluated participants who were diagnosed with liver failure and had undergone CMV DNA tests between January 2011 and April 2020. Consecutive hospitalized patients with liver failure were identified using the relevant International Classification of Diseases codes. Patients were only included if all the following criteria were met: (1) age between 18 and 80 years; (2) patients with an underlying liver failure diagnosis either at admission or during hospitalization; (3) patients with test results for CMV viral load. Patients were excluded if any of the following criteria were met: (1) transplant recipients; (2) CMV antibody IgG-negative; (3) CMV antibody IgM-positive; (4) missing key data for evaluation of disease severity and liver failure diagnosis; (5) died or discharged within 24 hours after admission; (6) patients with no follow-up records. A detailed flowchart for patient selection is presented in **Figure 1**.

Reactivation of latent CMV infection was defined as positive CMV DNA detected during hospitalization, with a previous positive CMV IgG result. Diagnostic criteria for liver failure were based on the Asian Pacific Association for the Study of the Liver criteria, that was International normalized ratio higher than 1.5 and total bilirubin higher than 5 times upper limits of normal (Shiv et al., 2019). Each patient was considered only once for demographic variables, evaluation of liver failure, and calculation of survival. Since no patient’s privacy data was showed, informed consent is not required. The study received ethical approval from the Institutional Review Board of Huashan Hospital affiliated with Fudan University.

### Data Collection and Glucocorticoid Treatment

Clinical and laboratory data were obtained from medical records. Symptoms were recorded at the time of CMV diagnosis. Laboratory results were obtained from the day of CMV reactivation. When data were not available for the same day, laboratory values within 3 days were used. Model for end‐stage liver disease (MELD) scores were then calculated.

Treatment history was collected and the use of immunosuppressant was of particular concern. Glucocorticoid treatment history was obtained, including drug, dose, and timing of administration prior to and during index admission. Glucocorticoids used three months prior to diagnosis were considered according to the methods described by a previous study (Gardiner et al., 2019). Glucocorticoid doses were converted to methylprednisolone equivalents, and average daily doses were calculated to allow for easier comparison between patients. Glucocorticoid regimens were also categorized as maintenance (long-term lower dose regimens typically comprising methylprednisolone dosage of 4–8 mg/day) or taper (shorter, higher intensity courses weaned down over a period of days to weeks).

### Control Selection and Follow‐Up

Cases were identified based on positive results for CMV viral load and an underlying diagnosis of liver failure. Patients with liver failure, but without evidence of CMV reactivation, were matched at a 2:1 ratio as control cases. Potential controls were matched to cases based on age (within 8 years) and year of admission (as close as possible, but no more than 1 year before or after). Identical exclusion criteria were used for control cases; patients known to be CMV IgG antibody-negative were also excluded. All suitable study participants were subjected to follow-up for at least two months.

### CMV DNA Test

CMV DNA viral load testing was performed using standard techniques. DNA was isolated from peripheral venous blood (3 mL), and CMV qPCR was performed using the CMV DNA Real Time PCR Kit (DA AN Gene Co., Ltd, Guangdong, CN) and the 7500 Real-Time PCR System (Bio-Rad). The limit of detection by qPCR was 500 copies/mL.

### Statistical Analysis

Categorical data were summarized as proportions and compared using the Chi-squared test or the Fisher’s exact test, as appropriate. Continuous variables were summarized as median and interquartile range (IQR), because their distributions were non-parametric, and compared using the Mann-Whitney *U* test. Univariate and multivariate analysis were used to explore risk factors for CMV reactivation. The Kaplan-Meier method was used to estimate survival probability, and the log-rank test was used to compare differences. Statistical analysis was performed using the statistical software GraphPad Prism (version 8.0; GraphPad Software, Inc.) and SPSS (version 23.0; IBM Corp, Chicago, IL, USA). All *P* values were two-sided, and values below 0.05 were considered statistically significant.

## Results

### Clinical Characteristics of Liver Failure Patients With and Without CMV Reactivation

Between January 2011 and April 2020, 209 adult patients with liver failure had CMV DNA test results. However, eleven patients were excluded (one with CMV antibody IgG-negative, two aged above 80 years of age, four died or discharged within 24 hours after admission, two with missing key data for liver failure diagnosis, and two with missing medical history or no follow-up), and the remaining one hundred ninety-eight patients were enrolled into the study (**Figure 1**). Among them, 33 patients had detectable CMV DNA in the plasma (16.7%), and they were included in the CMV-positive group.

he demographic and clinical characteristics of 198 patients with and without CMV reactivation are summarized in **Table 1**. In the CMV-positive group, the proportion of male was higher than that of the CMV-negative group (80.0% *vs* 60.6%, *P*=0.016). The proportion of patients in the CMV-positive group with an acute disease course was significantly higher than that in the CMV-negative group (42.4% *vs* 19.4%, *P*=0.007). One hundred thirty-six (82.4%) patients in the CMV-negative group presented with liver failure caused by hepatotropic virus infection, compared to 45.5% of the patients in the CMV-positive group (*P*=0.000).

**Table 1:** Demographic and clinical characteristics of 198 patients in liver failure with and without CMV reactivation.

Characteristic	Total (n=198)	CMV-Positive (n=33)	CMV-Negative (n=165)	*P* value
Age, median (IQR), y	49.5(37.0-60.0)	51.0(35.5-63.5)	49.0(37.5-59.5)	0.462
Male sex, n (%)	152(76.8)	20(60.6)	132(80.0)	**0.016**
Course of liver failure, n (%)				
Acute	46(23.2)	14(42.4)	32(19.4)	**0.007**
Acute on chronic	138(69.7)	17(51.5)	121(73.3)	**0.021**
Chronic	14(7.1)	2(6.1)	12(7.3)	0.999
Causes of liver failure, n (%)				
HAV/HBV/HCV/HEV	151(76.3)	15(45.5)	136(82.4)	**0.000**
Drug-induced	18(9.1)	5(15.2)	13(7.9)	0.190
Autoimmune	10(5.1)	5(15.2)	5(3.0)	**0.013**
Alcohol-induced	3(1.5)	3(9.1)	0	–
Wilson disease	12(6.1)	2(6.1)	10(6.1)	0.999
Other causes	4(2.0)	3(9.1)	1(0.6)	–
Underlying diseases, n (%)*				
Diabetes	11/166(6.6)	2(6.1)	9/133(6.8)	0.884
Hypertension	18/166(10.8)	4(12.1)	14/133(10.5)	0.792
Tumor	6/166(3.6)	2(6.1)	4/133(3.0)	0.400
Connective tissue diseases	3/166(1.8)	2(6.1)	1/133(0.8)	–
Hematological Diseases	10/166(6.0)	4(12.1)	6/133(4.5)	0.100
Co-infections	80(40.4)	19(57.6)	61(42.4)	**0.033**
Glucocorticoid use, n (%)				
Any glucocorticoids	63(31.8)	16(48.5)	47(28.5)	**0.024**
Total dose, median (IQR), mg	208.0(101.3-618.0)	836.5(308.7-1259.0)	160.0(80.0-353.0)	**0.000**
Taper glucocorticoids	28(14.1)	14(42.4)	14(8.9)	**0.000**
Maintenance glucocorticoids	14(7.1)	5(15.2)	9(5.5)	0.062
Any IV glucocorticoids	49(24.7)	14(42.4)	35(21.2)	**0.010**
Total dose, median (IQR), mg	384.0(107.8-996.3)	675.8(161.2-1110.0)	90.0(23.1-301.0)	**0.020**

Data are presented as No. (%), or median (interquartile range, IQR). HAV, Hepatitis A Virus; HBV, Hepatitis B Virus; HCV, Hepatitis C Virus; HEV, Hepatitis E Virus. IV, Intravenous.

*Thirty-two cases of the CMV negative group lacked a history of comorbidities so the data of this part contained a total of 166 patients. Bold values indicated that P values were significantly different between two groups.

Following the described 2:1 ratio, 66 control patients were matched based on age and year of admission. Various concomitant diseases included underlying conditions, such as diabetes, hypertension, tumor, connective tissue diseases, and hematological diseases, but there were no significant differences between two groups. Among 99 liver failure patients with or without CMV reactivation, there were no significant differences in liver failure manifestations, such as fever, fatigue, nausea, vomit, abdominal distension, jaundice, liver palms, spider nevus, ascites, lymphadenopathy, hepatomegaly, liver cirrhosis, splenomegaly, and hepatic encephalopathy (**Supplementary Table 1**).

### Laboratory Results for Diagnosis of CMV Reactivation

A comparison of laboratory findings in 99 liver failure patients with or without CMV reactivation is shown in **Table 2**. The serum albumin level in the CMV-positive group was significantly lower than that in the CMV-negative group (30 g/L *vs* 32.5 g/L, *P*=0.039). Interestingly, the median white cell count in the CMV-positive group was significantly higher than that in the CMV-negative group (10.1×10^9^ cells/L *vs* 6.0×10^9^ cells/L, *P*=0.001). In case of the lymphocyte subsets, the percentage of CD8+ T lymphocytes in the CMV-positive group was significantly higher than that in the CMV-negative group, and the absolute counts were relatively higher. On the contrary, the percentage of B lymphocytes and the absolute B lymphocyte count in the CMV-positive group were significantly lower than those in the CMV-negative group (*P*=0.018, *P*=0.036). There was no significant difference in other laboratory tests, including transaminases, coagulation function, hemoglobin, platelets, erythrocyte sedimentation rate (ESR), C-reactive protein, procalcitonin, ferritin, and lactic acid levels, between patients with and without CMV reactivation. The median MELD scores were almost equal between the two groups.

**Table 2:** Laboratory findings at diagnosis of CMV reactivation of 99 patients.

Variables	Total (n=99)	CMV-Positive (n=33)	CMV-Negative (n=66)	*P* value
**Alanine aminotransferase (ALT), IU/L**	143.0(69.0-408.0)	114.0(56.0-289.5)	152(69.8-534.5)	0.221
**Aspartate aminotransferase (AST), IU/L**	144.0(75.0-304.0)	146.0(81.5-282.5)	137.5(70.3-391.5)	0.953
**Alkaline phosphatase (ALP), IU/L**	144.0(111.0-186.0)	145.0(106.5-188.5)	145.0(115.8-183.0)	0.950
**Glutamyl transpeptidase (GGT), IU/L**	83.0(52.0-141.0)	108.0(57.0-186.0)	75.0(51.8-129.0)	0.158
**Total bilirubin, mg/dL**	297.5(203.5-423.7)	330.9(256.4-488.7)	284.7(176.9-399.7)	0.091
**Serum albumin, g/dL**	32.0(29.0-35.0)	30.0(27.0-34.0)	32.5(29.0-36.0)	**0.039**
**Serum globulin, g/dL**	29.0(22.8-35.3)	25.0(22.0-34.5)	29.0(23.0-35.5)	0.389
**International normalized ratio**	1.88(1.6-2.2)	1.9(1.6-2.2)	1.9(1.7-2.3)	0.427
**Prothrombin time, s**	21.1(17.9-25.3)	20.2(17.4-23.2)	21.6(17.9-25.6)	0.280
**Serum Creatinine, μ mol/L**	64.0(50.0-85.0)	62.0(48.5-98.5)	65.5(49.8-78.3)	0.944
**White cell counts, ×10^9^ cells/L**	7.1(4.7-10.3)	10.1(6.5-13.6)	6.0(4.5-8.7)	**0.001**
**Hemoglobin, g/L**	115.5(92.8-126.3)	105.0(90.0-124.0)	118.0(98.0-131.0)	0.091
**Platelet counts, ×10^9^ cells/L**	85.5(56.0-138.0)	80.0(44.5-145.5)	92.0(67.0-132.5)	0.441
**CD4+ T lymphocytes, %**	39.5(30.0-44.9)	37.0(29.4-44.0)	40.0(30.5-45.9)	0.454
**CD8+ T lymphocytes, %**	30.0(22.8-39.4)	35.0(23.7-45.8)	28.6(21.2-35.4)	**0.037**
**CD4+/CD8+ T lymphocytes**	1.3(0.9-2.0)	1.2(0.7-1.8)	1.3(0.9-2.0)	0.091
**B lymphocytes, %**	12.0(8.5-19.8)	9.4(4.8-13.5)	14.4(9.4-20.6)	**0.018**
**NK cell, %**	8.8(4.8-15.6)	7.9(2.0-15.5)	8.9(5.8-16.0)	0.172
**Absolute CD4+ T lymphocyte count,/μL**	298.4(167.8-426.0)	234.5(159.4-431.7)	312.3(169.4-424.2)	0.626
**Absolute CD8+ T lymphocyte count,/μL**	217.5(135.0-390.9)	220.5(140.9-541.4)	214.5(125.2-384.7)	0.536
**Absolute B lymphocyte count,/μL**	85.5(50.1-165.1)	70.3(26.6-141.6)	91.6(59.3-182.8)	**0.036**
**Absolute NK cell count,/μL**	70.7(26.5-159.8)	46.1(13.5-149.3)	80.2(37.5-161.4)	0.166
**ESR, mm/h**	4.0(2.0-18.8)	3.5(2.0-22.5)	4.0(2.0-18.8)	0.904
**CRP, mg/L**	11.9(6.6-23.9)	12.5(6.8-37.7)	10.4(6.3-21.7)	0.296
**PCT, ng/mL**	0.7(0.4-1.3)	0.7(0.4-1.9)	0.8(0.5-1.2)	0.652
**Ferritin, ng/mL**	1881.0(8944-2000)	2000.0(576-2000)	1695.0(1049-2000)	0.858
**Lactic acid, mmol/L**	1.7(1.2-2.7)	2.1(1.3-3.7)	1.7(1.1-2.6)	0.454
**MELD scores**	21.6(18.6-25.0)	21.7(17.4-26.7)	21.7(19.1-25.0)	0.678

Data are presented as median (interquartile range, IQR). ESR, Erythrocyte sedimentation rate; CRP, C reactive protein; PCT, procalcitonin; MELD, Model for end‐stage liver disease. Bold values indicated that P values were significantly different between two groups.

### Impact of CMV Reactivation on the Fatal Outcome of Patients With Liver Failure

**Figure 2:**
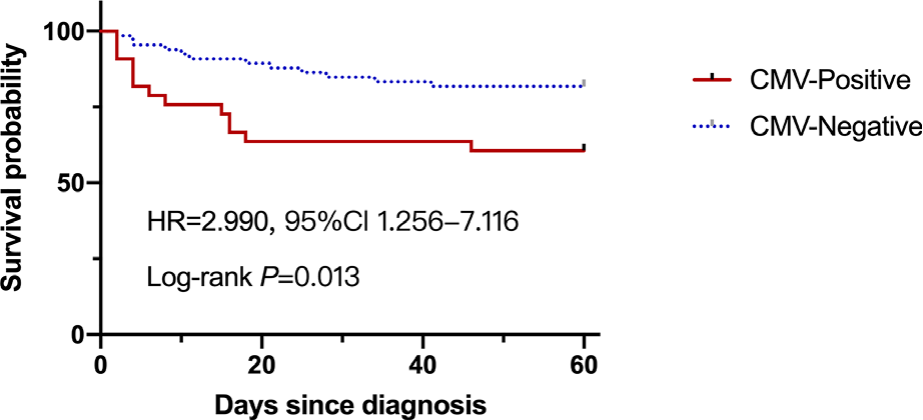
The survival probability of the liver failure patients with or without CMV reactivation at 60-day.

Overall mortality was 25.3% in the entire study at 60‐day from admission, which was significantly higher in the CMV-positive group compared to that in the CMV-negative group (39.4% *vs* 18.2%, *P*=0.028). As shown in **Figure 2**, the survival probability between two groups was significantly different, with nearly three times higher results in CMV-negative group compared to that in the CMV-positive group (HR=2.990, 95%Cl 1.256-7.116). We further explored the risk factors that contributed to fatal outcome in these patients and found that age, International normalized ratio, Prothrombin time, White cell counts and MELD scores were factors in fatal outcome, but Glucocorticoid use was not (**Supplementary Table 2**). Several typical factors were selected for a logistic regression analysis and a multivariate analysis showed that CMV reactivation was significantly associated with fatal outcome (adjusted *OR*, 5.33; 95% CI, 1.61–17.68; *P*=0.006), as showed in **Table 3**.

**Table 3:** Multivariate analysis: adjusted OR for risk of fatal outcome in liver failure patients.

	Fatal outcome	Benign outcome	Univariate analysis		Multivariate analysis	
**Variables**	**(n=25)**	**(n=74)**	**OR (95%Cl)**	***P* value**	**OR (95%Cl)**	***P* value**
**Age**	64(53-66)	49(36-60)	/	**0.000**	1.11(1.05-1.18)	**0.000**
**CMV reactivation**	13(52.0)	20(27.0)	2.93(1.16-7.47)	**0.000**	5.33(1.61-17.68)	**0.006**
**International normalized ratio**	2.1(1.7-3.3)	1.9(1.6-2.1)	/	**0.001**	3.79(1.49-9.67)	**0.005**
**MELD scores**	24.0(20.7-32.9)	21.3(18.5-24.0)	/	**0.018**	1.00(0.92-1.09)	0.967

Data are presented as No. (%), or median (interquartile range, IQR). MELD, Model for end‐stage liver disease. Bold values indicated that P values were significantly different between two groups.

### Glucocorticoid Regimens in Liver Failure Patients With and Without CMV Reactivation

There were 25 (25.3%) patients who reported usage of glucocorticoids of any type within 3 months prior to the CMV DNA test. The proportion of patients receiving glucocorticoids in the CMV-positive group was nearly 50%, compared to 13.6% in the CMV-negative group (*P*=0.000). The median total dose of glucocorticoids during the 3 months preceding CMV reactivation date comprised a total methylprednisolone dosage of 836.5 mg (IQR 308.7-1259.0 mg) in the CMV-positive group, which was significantly higher than that in the CMV-negative group (total methylprednisolone dosage of 280 mg, *P*=0.004). Specifically, 14 (42.4%) patients received a tapering course during the preceding 3 months in the CMV-positive group, which was markedly higher than that in the CMV-negative group (*P*=0.000). The proportions and dosages of intravenous glucocorticoid use in the CMV-positive group were higher than those in the CMV-negative group. However, the proportion of patients on maintenance glucocorticoid therapy in the two groups was not significantly different, as shown in **Table 1**, **Supplementary Table 3**.

### Risk Factors for CMV Reactivation in Liver Failure

To identify independent predictive factors for CMV reactivation in liver failure patients, we selected several typical factors for a logistic regression analysis (**Table 4**). A multivariate analysis revealed that liver failure caused by hepatitis virus was significantly associated with CMV reactivation (adjusted OR, 0.31; 95% CI, 0.11–0.88; *P*=0.027). Additionally, glucocorticoid use significantly increased the risk of CMV reactivation (adjusted OR, 4.84; 95% CI, 1.61–14.49; *P*=0.005). Patients with CMV reactivation tended to be associated with higher white cell counts, compared to patients without reactivation (adjusted OR, 1.21; 95% CI, 1.08–1.36; *P*=0.002). These results suggest that patients with liver failure caused by hepatitis virus may be less predisposed to CMV reactivation; however, use of glucocorticoids may be a risk factor. Thus, when white cell counts are elevated, CMV reactivation needs to be considered.

**Table 4:** Multivariate analysis: adjusted OR for risk of CMV reactivation in liver failure patients.

	CMV-Positive	CMV-Negative	Univariate analysis		Multivariate analysis	
**Variables**	**(n=33)**	**(n=66)**	**OR (95%Cl)**	***P* value**	**OR (95%Cl)**	***P* value**
**Cause by HAV/HBV/HCV/HEV**	15(45.5)	52(78.8)	0.22(0.09-0.56)	**0.001**	0.31(0.11-0.88)	**0.027**
**Any glucocorticoids used**	16(48.5)	9(13.6)	5.96(2.24-15.88)	**0.000**	4.84(1.61-14.49)	**0.005**
**White cell counts, ×10^9^ cells/L**	10.1(6.5-13.6)	6.0(4.5-8.7)	/	**0.001**	1.21(1.08-1.36)	**0.002**

HAV, Hepatitis A Virus; HBV, Hepatitis B Virus; HCV, Hepatitis C Virus; HEV, Hepatitis E Virus. Bold values indicated that P values were significantly different between two groups.

## Discussion

While CMV reactivation is a known major cause of morbidity and mortality in transplant recipients, its role as a pathogen in patients with transplant-free liver failure is not well understood. In this study, we showed that CMV reactivation occurred at a high rate (16.7%) in patients with liver failure. Clinical manifestations and liver functions were comparable between the CMV reactivation and non-reactivation groups, resulting in neglected virologic surveillance. However, CMV reactivation may triple mortality in patients with liver failure, thereby demonstrating its important effect on prognosis. Similar to findings of previous studies in other populations, such as patients with rheumatic diseases, we found that the use of high glucocorticoid doses, especially those administered intravenously, might lead to CMV reactivation (Gardiner et al., 2019; Li et al., 2019). To the best of our knowledge, this is the first report to investigate the risk factors of CMV reactivation and their effect on prognosis in patients with transplant-free liver failure. Our study supports the role of CMV virologic surveillance in liver failure.

CMV reactivation has been recognized as a cause of poor prognosis in other patient populations, such as those with hematological malignancies, HSCT, HIV infection, and rheumatic diseases (Strippoli et al., 2006; Fujimoto et al., 2013; Hardie et al., 2013). Furthermore, CMV reactivation was an independent predictor of pneumocystis pneumonia in solid organ transplant recipients, in association with a poor prognosis (Hosseini-Moghaddam et al., 2018). However, the risk factors and outcomes of CMV reactivation in liver failure patients remain to be extensively investigated. Previous study indicated that CMV coinfection was a possible risk factor for the progression of liver fibrosis (Ibrahim et al., 2017). A cytokine storm may be induced, leading to the development of sepsis in patients with liver failure, who are susceptible for opportunistic pathogens such as CMV (Sarin and Choudhury, 2018). In the present study, CMV reactivation occurred in liver failure patients at a high rate of more than 15%, which was higher than that reported in a previous study in China (Hu et al., 2018). The higher rate may be due to the fact that the previous study focused on liver failure caused by Hepatitis B virus, while our study included liver failure with multiple underlying causes. We indicated that liver failure caused by hepatotropic virus infection, such as Hepatitis B, seemed to be less prone to CMV reactivation, compared to other causes. Actually, compared to the patients with liver failure caused by drug-induced or autoimmune, who may receive glucocorticoids, the patients with liver failure caused by hepatitis virus showed lower proportion with CMV reactivation. Noteworthy, both clinical manifestations and liver function were not evidently different between the CMV reactivation and non-reactivation groups, which resulted in neglected CMV detection. In our retrospective study, CMV DNA tests results were unavailable in one third of all patients. It was unclear whether the low numbers of CMV-positive patients were due to the rarity of this combination of events, or because of under-diagnosis owing to the lack of testing in this population.

Immunosuppression is a widely recognized risk factor for CMV reactivation (Tong et al., 2013). Consistent with the published literature, the use of high doses of glucocorticoids was found to contribute to CMV reactivation in liver failure patients (Choo et al., 2019). Furthering previous studies, we explored the effects of different glucocorticoid doses and forms on CMV reactivation. We found that the dosage of glucocorticoids in the CMV-positive group was significantly higher than that in the CMV-negative group. It seemed that the use of intravenous glucocorticoids was more likely to reactivate CMV, while oral glucocorticoid intake with a low dose maintenance had no effect on CMV reactivation (Gardiner et al., 2019). In the present study, CMV reactivation was less frequent in patients with hepatotropic virus infection, which might be associated with lower use of glucocorticoids in liver failure caused by viral hepatitis. Furthermore, a novel finding of the current study is the association of higher white blood cell counts with CMV reactivation in liver failure patients, but such an association may be secondary to a higher dose of glucocorticoids or coinfection with bacteria in CMV reactivation patients. As shown in **Table 1**, the co-infection rate in the CMV-positive group was significantly higher than that in the CMV-negative group (57.6% *vs* 42.4%, *P*=0.033), which confirmed that immunosuppression would lead to co-infections with opportunistic pathogens. CMV reactivation through NF-κB mediated activation has been found to be linked to co-infection by pathogens through the inflammation, DNA damage and oxidative stress notably (Hummel and Abecassis, 2002; Liu et al., 2016). By inhibiting the NF-κB signaling pathway, co-infection following CMV reactivation will be alleviated (Chen et al., 2018).

The composition of lymphocyte subsets can reflect the immune status of an individual to some extent. In the present study, we explored the changes in lymphocyte subsets in the study population and found that the percentage of CD8^+^ T lymphocytes in the CMV-positive group was significantly higher than that in the CMV-negative group, and that the absolute counts were relatively higher. The results were plausible, because CD8^+^ T cells play a major role in the control of CMV viremia (Attaf et al., 2020). A previous study showed that patients with low B cell counts before HSCT might be at a risk of developing infections (Gernert et al., 2020). Sepsis non-survivors exhibited reduced B cell numbers at the onset of sepsis, compared to sepsis survivors (Krautz et al., 2018). In concordance, comparison of CMV reactivation patients with non-reactivation patients showed significant differences in B cell percentages and B cell numbers in our study. The percentages of B lymphocytes and B cell numbers in the CMV-positive group were significantly lower than those in the CMV-negative group. This result suggested that low B cell counts were associated with a higher possibility of CMV reactivation, which necessitates careful monitoring of patients with low B cell numbers.

There were several limitations in this study. First, this was a single-center, retrospective study with statistical power limited by the sample size. Despite extending our search over a 10-year study period, we could only identify 33 patients with liver failure and proven CMV reactivation for comparison. A further prospective multicenter study with a larger sample size is required for validation. Second, although we enrolled patients who were CMV IgG antibody-positive and demonstrated detectable CMV DNA as CMV reactivation cases, we could not ascertain reactivation in all cases, apart from confirmation of primary disease, because we could have missed the time window when IgG status remained negative for primary disease. However, given the known high seroprevalence rate of CMV, particularly amongst older adults, we assumed that most patients were likely to be CMV seropositive from childhood.^1^ Third, we only performed qualitative detection of CMV DNA; hence, we could not analyze the relationship between CMV DNA load and severity of prognosis. PCR methodology will be improved, and we will address this issue in our future studies. Finally, quantification of glucocorticoid use was difficult, as steroid regimens could be complex and often change with varied responses of patients to therapy. However, after examination of glucocorticoid usage in multiple ways, it was evident that CMV reactivation cases had received significantly higher amounts compared to those received by controls.

In conclusion, we found that CMV reactivation was associated with mortality in liver failure. High glucocorticoid doses intravenously seemed to be the most important risk factor for CMV reactivation in liver failure. Thus, monitoring CMV DNA dynamically may be considered when using glucocorticoids in patients with liver failure. Once CMV DNA is identified, early intervention by therapeutic antivirals may improve liver failure prognosis.

## Data Availability Statement

The original contributions presented in the study are included in the article/**Supplementary Material**. Further inquiries can be directed to the corresponding authors.

## Ethics Statement

The studies involving human participants were reviewed and approved by Institutional Review Board of Huashan Hospital affiliated with Fudan University. Since no patient’s privacy data was showed, informed consent was not required.

## Author Contributions

Conception or design of the work (QY, LS, and JZ). Execution of CMV DNA viral load testing (YC and AL). Statistical analysis and interpretation of data (QY, ZZ, XY, and BZ). Drafting the manuscript (QY, ZZ, and XY). Accountability for all aspects of the work and final approval of the version to be submitted (LS, JZ, and WZ). All authors contributed to the article and approved the submitted version.

## Funding

This work was supported by research grants from the Natural Science Foundation of Shanghai, China (grant number 19ZR1407800) and the Key Laboratory Project of Shanghai Science and Technology Commission [grant numbers 20dz2210400].

## Conflict of Interest

The authors declare that the research was conducted in the absence of any commercial or financial relationships that could be construed as a potential conflict of interest.
