# Supplementary material for: Latent Cytomegalovirus Reactivation in Patients With Liver Failure: A 10-Year Retrospective Case-Control Study, 2011-2020
Source: Front Cell Infect Microbiol. 2021 May 10;11:642500. doi: 10.3389/fcimb.2021.642500 (PMC8143188; doi:10.3389/fcimb.2021.642500)
Supplement: Supplementary file 1 [file Table_1.docx]

**Supplementary Table 1. Manifestations of liver failure patients with or without CMV reactivation.**

| Characteristic | Total  (n=99) | | CMV-Positive  (n=33) | | CMV-Negative  (n=66) | | *P* value |
| --- | --- | --- | --- | --- | --- | --- | --- |
| Fever | 34(34.3) | 14(42.4) | | 20(30.3) | | 0.231 | |
| Fatigue | 90(90.9) | 31(93.9) | | 59(89.4) | | 0.458 | |
| Nausea, poor appetite | 80(80.8) | 27(81.8) | | 53(80.3) | | 0.857 | |
| Vomit | 15(15.2) | 7(21.2) | | 8(12.1) | | 0.234 | |
| Abdominal distension | 33(33.3) | 10(30.3) | | 23(34.8) | | 0.651 | |
| Jaundice | 98(99.0) | 33(100.0) | | 65(98.5) | | 0.477 | |
| Liver palms | 27(27.3) | 11(33.3) | | 16(24.2) | | 0.338 | |
| Spider nevus | 10(10.1) | 4(12.1) | | 6(9.1) | | 0.637 | |
| Ascites | 42(42.4) | 17(51.5) | | 25(37.9) | | 0.196 | |
| Lymphadenopathy | 10(10.1) | 5(15.2) | | 5(7.6) | | 0.238 | |
| Hepatomegaly | 4(4.0) | 3(9.1) | | 1(1.5) | | 0.071 | |
| Liver cirrhosis | 35(35.4) | 14(42.4) | | 21(31.8) | | 0.298 | |
| Splenomegaly | 44(44.4) | 15(45.5) | | 29(43.9) | | 0.886 | |
| Hepatic encephalopathy | 18(18.2) | 7(21.2) | | 11(16.7) | | 0.580 | |

Data are presented as No. (%).

**Supplementary Table 2. Variables compared between patents of liver failure with and without fatal outcome in univariate analysis.**

| **Variables** | ***P* value** |
| --- | --- |
| Age | **0.000** |
| CMV reactivation | **0.022** |
| Male sex | 0.058 |
| Course of liver failure | 0.088 |
| Causes of liver failure | 0.471 |
| Diabetes | 0.716 |
| Hypertension | 0.258 |
| Tumor | 0.436 |
| Connective tissue diseases | 0.307 |
| Hematological Diseases | 0.558 |
| Glucocorticoid use | 0.868 |
| Alanine aminotransferase (ALT), IU/L | 0.732 |
| Aspartate aminotransferase (AST), IU/L | 0.787 |
| Alkaline phosphatase (ALP), IU/L | 0.219 |
| Glutamyl transpeptidase (GGT), IU/L | 0.088 |
| Total bilirubin, mg/dL | 0.416 |
| Serum albumin, g/dL | 0.272 |
| Serum globulin, g/dL | 0.114 |
| International normalized ratio | **0.032** |
| Prothrombin time, s | **0.020** |
| Serum Creatinine, umol/L | 0.089 |
| White cell counts, ×10^9^ cells/L | **0.036** |
| Hemoglobin, g/L | 0.605 |
| Platelet counts, ×10^9^ cells/L | 0.912 |
| CD4+ T lymphocytes, % | 0.433 |
| CD8+ T lymphocytes, % | 0.254 |
| CD4+/ CD8+ T lymphocytes | 0.398 |
| B lymphocytes, % | 0.521 |
| NK cell, % | 0.594 |
| Absolute CD4+ T lymphocyte count, /μL | **0.007** |
| Absolute CD8+ T lymphocyte count, /μL | **0.003** |
| Absolute B lymphocyte count, /μL | **0.041** |
| Absolute NK cell count, /μL | 0.202 |
| ESR, mm/h | 0.744 |
| CRP, mg/L | 0.802 |
| PCT, ng/mL | 0.229 |
| Ferritin, ng/mL | **0.017** |
| Lactic acid, mmol/L | **0.076** |
| MELD scores | **0.018** |

Noted: Bold values indicated that *P* values were significantly different between two groups.
**Supplementary Table 3. Glucocorticoid treatment of 99 patients in liver failure with and without CMV reactivation.**

| **Characteristic** | **Total**  **(n=99)** | **CMV-Positive**  **(n=33)** | **CMV-Negative**  **(n=66)** | ***P* value** |
| --- | --- | --- | --- | --- |
| Any glucocorticoids | 25(25.3) | 16(48.5) | 9(13.6) | **0.000** |
| Total dose, median (IQR), mg | 580.0(161.0-1056.0) | 836.5(308.7-1259.0) | 280.0(70.0-360.0) | **0.004** |
| Taper glucocorticoids | 19(19.2) | 14(42.4) | 5(7.6) | **0.000** |
| Maintenance glucocorticoids | 8(8.1) | 5(15.2) | 3(4.6) | 0.113 |
| Any IV glucocorticoids | 20(20.2) | 14(42.4) | 6(9.1) | **0.000** |
| Total dose, median (IQR), mg | 384.0(107.8-996.3) | 675.8(161.2-1110.0) | 100.0(51.6-301.0) | **0.026** |

Data are presented as No. (%), or median (interquartile range, IQR). IV: Intravenous.
